# Association between triglyceride-glucose index and risk of colorectal carcinogenesis: a meta-analysis of observational studies

**DOI:** 10.3389/fonc.2025.1569824

**Published:** 2025-12-17

**Authors:** Lichao Wang, Yuxuan Lin, Yonghe Liao, Jinhai Shen

**Affiliations:** 1 Department of General Surgery, The Second Hospital of Hebei Medical University, Shijiazhuang, Hebei, China; 2 Department of Pharmacy, Guangxi Hospital Division of The First Affiliated Hospital, Sun Yat-sen University, Nanning, Guangxi, China; 3 School of Pharmaceutical Science, Guangxi Medical University, Nanning, Guangxi, China; 4 State Key Laboratory of Natural Medicines, China Pharmaceutical University, Nanjing, Jiangsu, China; 5 Center for New Drug Safety Evaluation and Research, China Pharmaceutical University, Nanjing, Jiangsu, China; 6 School of Basic Medicine and Clinical Pharmacy, China Pharmaceutical University, Nanjing, Jiangsu, China

**Keywords:** triglyceride-glucose index, colorectal carcinogenesis, surrogate marker, meta-analysis, risk factor

## Abstract

**Background:**

The relationship between the triglyceride-glucose (TyG) index and the risk of colorectal carcinogenesis, encompassing both colorectal adenomas and carcinoma, is not yet definitively established. This meta-analysis aims to synthesize available evidence to provide a comprehensive estimate of the association between the TyG index and the likelihood of this disease spectrum.

**Methods:**

A systematic search was conducted in PubMed, Embase, and Scopus to identify studies examining the TyG index and colorectal carcinogenesis risk. Meta-analysis was performed to calculate effect sizes (ESs) and their corresponding 95% confidence intervals (CIs) to obtain a summary estimate.

**Results:**

A total of nine observational studies involving 1,056,401 participants were included in the analysis. The meta-analysis revealed that a continuous increase in the TyG index was associated with an elevated risk of colorectal carcinogenesis (ES, 1.19; 95% CI, 1.05-1.34). Similarly, a one-unit increase in the TyG index was linked to an increased risk of colorectal carcinogenesis (ES, 1.24; 95% CI, 1.15-1.32). Additionally, compared to the lowest quartile, the second (ES, 1.07; 95% CI, 1.02-1.13), third (ES, 1.12; 95% CI, 1.06-1.18), and highest quartile (ES, 1.19; 95% CI, 1.13-1.24) of the TyG index exhibited a significantly higher risk of colorectal carcinogenesis.

**Conclusion:**

This meta-analysis demonstrates a significant association between the TyG index and colorectal carcinogenesis, suggesting that the TyG index could serve as a convenient and reliable surrogate marker for identifying individuals at risk. These findings may inform screening and prevention strategies targeting the full spectrum of colorectal neoplasia. Standardization of TyG index cutoffs and validation in diverse populations are warranted.

**Systematic review registration:**

https://www.crd.york.ac.uk/prospero/, identifier CRD42024605775.

## Introduction

Colorectal cancer (CRC) represents a major global health challenge, ranking among the leading causes of cancer-related mortality worldwide and imposing a substantial burden on public health systems (1, 2). Although genetic factors account for a minority of CRC cases, most are sporadic, arising from a complex interplay between environmental exposures and lifestyle factors (3). Accumulating evidence indicates that the majority of CRCs evolve from pre-existing colorectal adenomas through the well-established adenoma–carcinoma sequence, underscoring adenomas as critical precancerous lesions. Therefore, identifying reliable risk factors and developing effective preventive strategies for colorectal carcinogenesis remain of paramount importance.

Insulin resistance (IR), a metabolic disorder characterized by reduced responsiveness to insulin, has been increasingly recognized as a key factor in the pathogenesis of CRC (4, 5). The hyperinsulinemic-euglycemic glucose clamp technique is considered the gold standard for directly quantifying insulin sensitivity and estimating IR (6). However, this method is not only time-consuming and costly but also labor-intensive and technically complex, which limits its feasibility for large-scale epidemiological studies, particularly in resource-limited settings. The homeostatic model assessment of IR (HOMA-IR), on the other hand, offers an indirect means of evaluating IR through a mathematical model (7). However, its clinical utility is limited, as it requires the measurement of fasting insulin—an assessment that is not routinely performed in clinical practice unless there is a specific need to evaluate the endocrine system.

Given the challenges associated with both direct and indirect methods for assessing IR, there is a growing demand for more accessible and practical tools for its evaluation. In response, researchers have focused on developing surrogate markers that can simplify the screening process for IR. Among these, the triglyceride-glucose (TyG) index has emerged as a promising and practical surrogate marker (8). The TyG index is calculated using a simple formula: the natural logarithm of the product of fasting triglycerides and fasting plasma glucose levels divided by 2 (ln [fasting triglycerides (mg/dL) × fasting glucose (mg/dL)]/2). This approach offers significant advantages, as it bypasses the need for direct insulin measurement and presents a more feasible alternative to other indirect assessment methods. The simplicity and practicality of the TyG index have made it a focal point in colorectal carcinogenesis research, offering a more accessible means of identifying individuals at risk for colorectal carcinogenesis.

Colorectal carcinogenesis is a multistep process that encompasses both the formation of colorectal adenomas and their potential malignant transformation into CRC. Although several studies have explored the association between the TyG index and the risk of colorectal carcinogenesis, the results remain inconclusive. In light of this uncertainty, a comprehensive meta-analysis is warranted to integrate the available studies. Therefore, we conducted a meta-analysis to comprehensively assess the association between the TyG index and the risk of colorectal carcinogenesis, with the goal of informing future clinical practices and preventive strategies.

## Methods

### Protocol and guideline

This study was registered in the International Prospective Register for Systematic Reviews under the identifier CRD42024605775, and was conducted in accordance with the Preferred Reporting Items for Systematic Reviews and Meta-Analyses (PRISMA) statement (9).

### Data source and study selection

An exhaustive systematic search was performed in databases including PubMed, Scopus, Embase, Web of Science, and Cochrane Library. A combination of Medical Subject Headings terms and free texts was utilized to identify all studies published up to the 9th of November 2024. The search queries were constructed using the refined terms: ‘Triglyceride-Glucose Index’, ‘Colorectal Neoplasms’, and ‘Colorectal Cancer’. The details of searching strategies are presented in **Supplementary Table 1**. No filters based on language or publication status were applied during the initial search to maximize the comprehensiveness of our retrieval.

We included studies that (i) investigated the relationship between TyG index and colorectal carcinogenesis, including both colorectal adenomas and carcinoma, (ii) provided data on odds ratios (OR), relative risks (RR), or hazard ratios (HR). The following types of studies were excluded: (i) Reviews, (ii) case reports, and (iii) preclinical studies.

The literature was consolidated and duplicate entries were eliminated using EndNote. Following this, two independent researchers conducted an initial review of study titles and abstracts. Eligible studies were then subjected to a full-text evaluation. Any discrepancies between the reviewers were arbitrated by a third investigator.

### Data extraction and quality assessment

Data were collected and summarized by one investigator and reviewed by another reviewer. Any discrepancies were resolved by consensus between the reviewers. The subsequent details were collected and presented in a table: first author’s name, publication year, country of origin, study design, study population, sample size, details about the TyG index, mean age, proportion of males, along with the ORs, RRs, and HRs accompanied by their 95% confidence intervals (CI) for colorectal adenoma and/or carcinoma, when available.

The quality assessment was performed utilizing the Newcastle-Ottawa Scale (NOS) (10). Studies assessed with NOS scores between seven and nine were categorized as high-quality, while those scoring between five and six were classified as moderate-quality. Studies scoring four or below were regarded as low-quality.

### Statistical analysis

All statistical analyses were conducted using R (v4.3.2). A meta-analysis was performed to compare the association between continuous increase level in TyG index and the risk of colorectal carcinogenesis, including adenomas and carcinoma, yielding a combined effect size (ES) and its 95% CI. The impact of a one-unit increase in the TyG index on colorectal carcinogenesis risk was evaluated by calculating ORs, RRs, or HRs along with their 95% CIs through meta-analysis to obtain an overall estimate. Additionally, separate meta-analyses were conducted to assess the risk of colorectal carcinogenesis for each quartile of the TyG index relative to the lowest quartile.

The choice between a fixed-effects model and a random-effects model for producing combined ES depended on the level of heterogeneity observed. Heterogeneity was evaluated using the *I²* statistic and the Cochrane *Q* test, with significance defined as an *I²* exceeding 50% and a *Q* test p-value below 0.1. Publication bias was detected using funnel plots and Egger’s test. Sensitivity analyses, using the leave-one-out method, were conducted to evaluate the influence of individual studies on the pooled effect estimates. In this meta-analysis, OR, RR, and HR were considered equivalent in their interpretation. A two-tailed *P*-value less than 0.05 was considered statistically significant.

## Results

### Study selection

**Figure 1:**
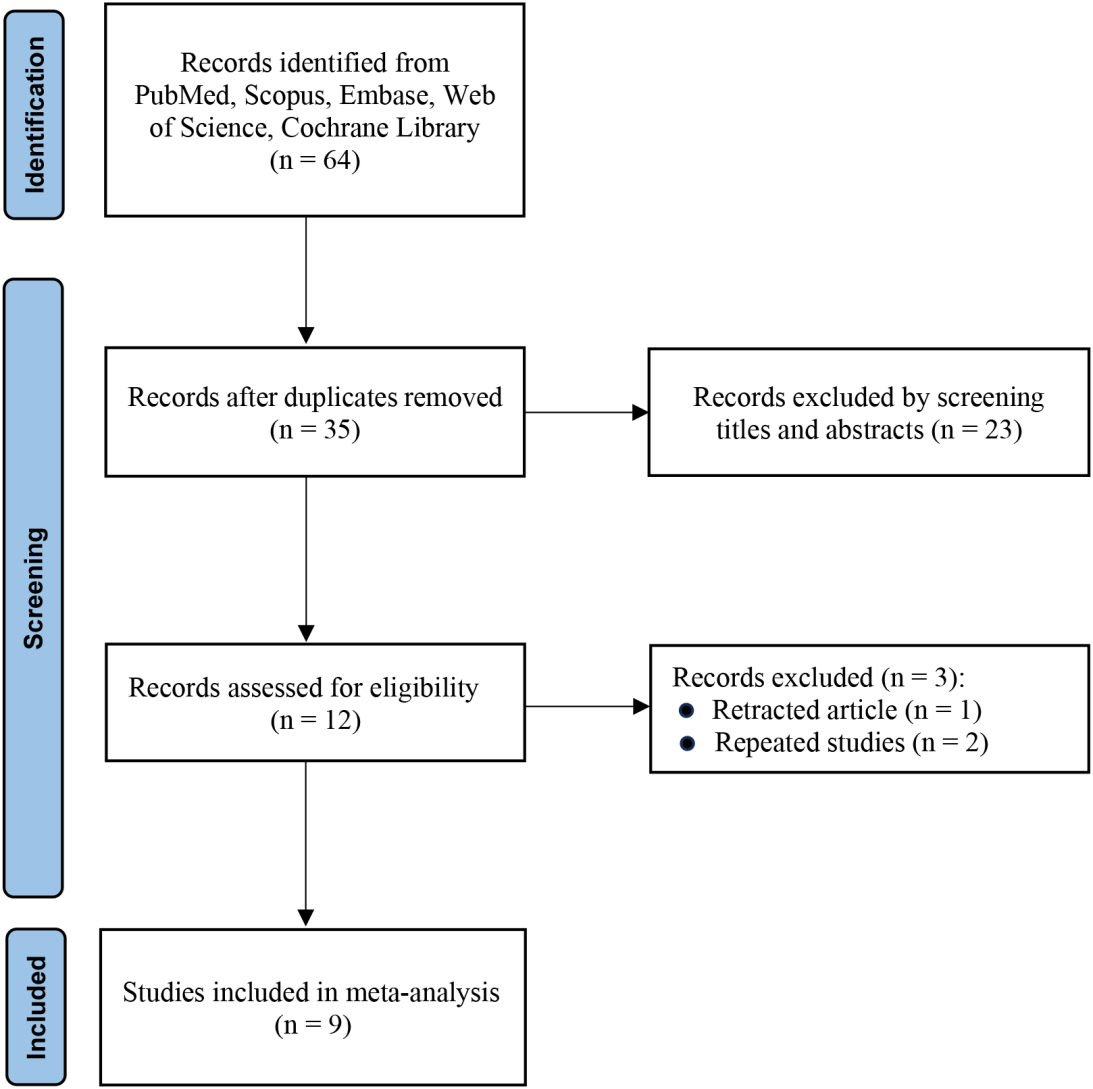
PRISMA flow diagram of study selection.

The initial search retrieved 64 records. After removing duplicates, 35 studies remained for preliminary screening. After carefully reviewing the titles and abstracts, 23 studies were considered irrelevant and thus excluded. Subsequently, 12 studies were subjected to more detailed evaluation. Eventually, a total of 9 studies that fulfilled all the eligibility requirements were included in the meta-analysis (11–19). The study selection process is illustrated in **Figure 1**.

### Characteristics of the included studies

Among the included studies, there was three cross-sectional studies (13, 14, 18), one case-control study (16), and five cohort studies (11, 12, 15, 17, 19), encompassing a total of 1,056,401 individuals. These studies were conducted between 2020 and 2024, spanning regions in East Asia (China, Japan, and South Korea) and Europe. The sample sizes of these studies varied from 1,472 to 510,471 participants. A higher proportion of males was observed among patients with colorectal neoplasms compared to the control group. Additionally, there was a trend of increasing male prevalence with higher levels of the TyG index. According to the NOS criteria for evaluating the quality of observational studies, all the included studies were of high quality, as detailed in **Supplementary Table 2**. The characteristics of the included studies are summarized in **Table 1**.

**Table 1:** Characteristics of studies included in meta-analysis.

Study	Country / Region	Design	Population	Sample size	TyG index information	Age ± SD, years	Male (%)	Outcomes reported	NOS
Son et al. 2024 (11)	Korea	Retrospective cohort study (follow-up: 9 to 10 years)	General population aged over 40	314,141	Categorized Q1: 8.0 ± 0.2 Q2: 8.5 ± 0.1 Q3: 8.8 ± 0.1 Q4: 9.4 ± 0.3	Q1: 57.8 ± 8.5	35,857 (45.6)	Reference	7
						Q2: 59.0 ± 8.8	39,296 (50.8)	HR (95% CI) 1.08 (1.00-1.16)	
						Q3: 59.4 ± 8.8	43,783 (55.5)	HR (95% CI) 1.10 (1.02-1.19)	
						Q4: 59.1 ± 8.7	50,396 (63.6)	HR (95% CI) 1.16 (1.07-1.25)	
Liu et al. 2022 (12)	China	Prospective cohort study (median follow-up: 13 years)	General adult population	93,659	Categorized Q1: ≤ 8.19 Q2: 8.19-8.58 Q3: 8.58-9.06 Q4: ≥ 9.06	All patients: 51.44 ± 12.45 Women: 48.67 ± 11.46 Men: 52.24 ± 12.60	74,671 (79.7)	Reference	7
								HR (95% CI) 1.13 (0.88-1.45)	
								HR (95% CI) 1.36 (1.06-1.76)	
								HR (95% CI) 1.50 (1.19-1.91)	
Li et al. 2024 (13)	China	Cross-sectional study	Adult participants who underwent complete colonoscopy at hospital	3,061	Categorized Q1: < 8.13 Q2: 8.13-8.50 Q3: 8.51-8.94 Q4: > 8.94	Q1: 55.7 ± 14.8	312 (40.3)	Reference	7
						Q2: 57.4 ±12.8	382 (50.2)	OR (95% CI) 1.32(1.02-1.72)	
						Q3: 56.6 ± 12.2	420 (55.7)	OR (95% CI) 1.35(1.03-1.77)	
						Q4: 54.0 ± 12.5	527 (68.3)	OR (95% CI) 1.45(1.06-1.98)	
Choi et al. 2024 (14)	Korea	Cross-sectional study	Individuals aged 20 to 49 years who underwent their first screening colonoscopy	4,467	Continuous Control: 8.32 ± 0.61 Case: 8.51 ± 0.71	All patients: 39.02 ± 6.32 Control: 38.41 ± 6.36 Case: 41.36 ± 5.58	All patients: 2,448 (54.8) Control: 1,841 (51.9) Case: 607 (66.0)	OR (95% CI) 1.16 (1.02-1.33)	7
Fritz et al. 2020 (15)	Europe	Retrospective cohort study (median follow-up: 17.2 years)	General population	510,471	Categorized Q1: < 8.1 Q2: 8.1-8.4 Q3: 8.4-8.7 Q4: 8.7-9.1 Q5 > 9.1	Q1: 39.6 ± 11.0	33,153 (32.3)	Reference	7
						Q2: 42.8 ± 10.7	41,317 (40.5)	HR (95% CI) Colon: 0.98 (0.88-1.10) Rectum: 1.04 (0.90-1.19)	
						Q3: 43.6 ± 10.7	49,640 (48.7)	HR (95% CI) Colon: 1.07 (0.96-1.19) Rectum: 1.12 (0.98-1.28)	
						Q4: 44.4 ± 10.2	59,811 (58.7)	HR (95% CI) Colon: 1.16 (1.04-1.29) Rectum: 1.13 (0.99-1.30)	
						Q5: 44.9 ± 9.4	74,047 (72.5)	HR (95% CI) Colon: 1.14 (1.03-1.27) Rectum: 1.24 (1.08-1.42)	
Han et al. 2022 (16)	China	Case-control study	Adult patients without cardiovascular disease underwent colonoscopy	2,409	Categorized Control: 8.63 ± 0.63 Case: 8.71 ± 0.60	All patients: 57.18 ± 11.26 Control: 54.04 ± 11.87 Case: 59.22 ± 10.36	All patients: 1,355 (56.25) Control: 457 (48.26) Case: 898 (61.42)	OR (95% CI) Q1: Reference Q2: 1.24 (0.94-1.63) Q3: 1.22 (0.93-1.60) Q4: 1.35 (1.02-1.77)	7
Okamura et al. 2020 (17)	Japan	Retrospective cohort study (median follow-up: 4.4 years)	General population	27,921	Continuous CRC-: 8.2 ± 0.7 CRC+: 8.4 ± 0.7	All patients: 45.7 ± 10.1 CRC-: 45.6 ± 10.1 CRC+: 51.1 ± 9.3	All patients: 16,434 (58.9) CRC-: 16,353 (58.8) CRC+: 81 (69.8)	HR (95% CI) 1.38 (1.00-1.91)	7
Lam et al. 2023 (18)	China Hong Kong	Cross-sectional study	Asymptomatic subjects aged between 50 and 75	1,472	Categorized Q1: < 6.64 Q2: 6.64-7.01 Q3: 7.01-7.40 Q4: > 7.40	Adenoma: 61.0 ± 6.44 No adenoma: 59.9 ± 5.23	Adenoma: 533 (66.6) No adenoma: 277 (43.1)	OR (95% CI) Q1: Reference Q2: 1.37 (0.99-1.90) Q3: 1.00 (0.72-1.90) Q4: 1.43 (1.02-2.00)	7
Kityo et al. 2024 (19)	Korea	Prospective cohort study (median follow-up: 10.6 years)	Participants aged 40-69 years	98,800	Categorized Q1: 7.7 ± 0 Q2: 8.3 ± 0 Q3: 8.6 ± 0 Q4: 9.3 ± 0	Q1: 50.2 ± 0.05 Q2: 52.1 ± 0.05 Q3: 53.3 ± 0.05 Q4: 53.3 ± 0.05	Q1: 4,792 (19.4) Q2: 6,742 (27.3) Q3: 8,869 (35.9) Q4: 12,642 (51.2)	HR (95% CI) 1.31 (1.14-1.52)	7

TyG, triglyceride-glucose; SD, standard deviation; NOS, Newcastle-Ottawa Scale; Q, quartile; HR, hazard ratio; OR, odds ratio; CI; confidence interval.

### Association between continuous increase in the TyG and risk of colorectal carcinogenesis

**Figure 2:**
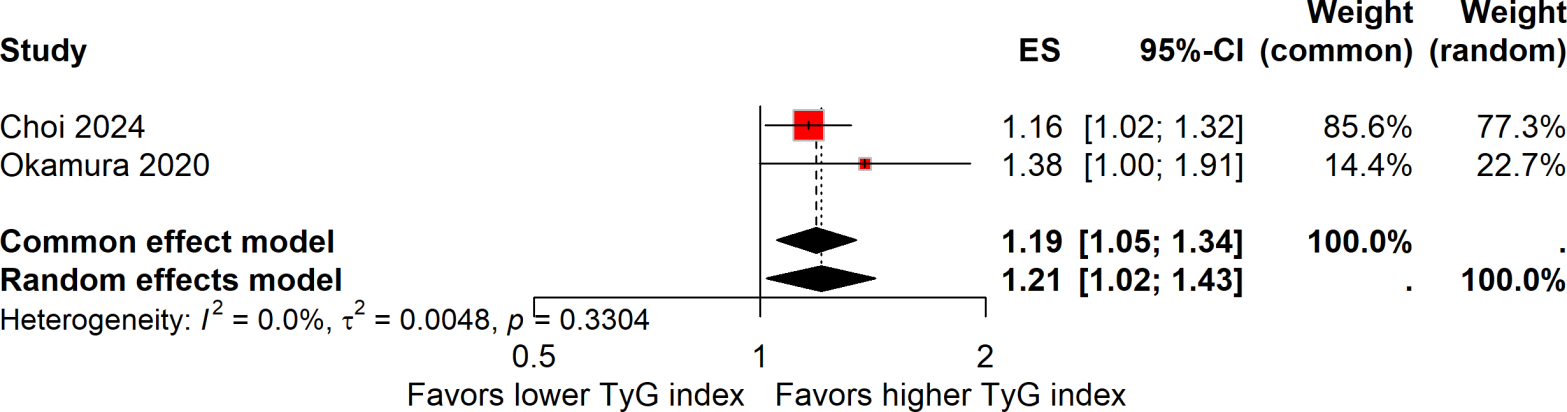
Forest plot showing the association between a continuous increase in the TyG index and the risk of colorectal carcinogenesis. Both common-effect (fixed-effect) and random-effects models were applied as a sensitivity analysis despite the absence of heterogeneity (*I²* = 0%). The weight of each study reflects precision rather than sample size.

The relationship between the continuous elevation of the TyG index and the risk of colorectal carcinogenesis was investigated in two studies, encompassing a total of 32,388 participants (14, 17). Despite its smaller sample size, Choi et al. contributed 85.6% of the overall weight because its effect estimate was more precise (OR 1.16, 95% CI 1.02–1.33) than that of Okamura et al. (HR 1.38, 95% CI 1.00–1.91). Given the absence of heterogeneity (*I²* = 0%), a fixed-effect model was used to calculate the pooled estimate, and a random-effects model was additionally performed as a sensitivity check showing consistent results. Overall, the meta-analysis showed that a continuous increase in the TyG index was associated with a 19% higher risk of colorectal carcinogenesis (ES, 1.19; 95%CI, 1.05-1.34; **Figure 2**).

### Impact of a one-unit increment in the TyG index on colorectal carcinogenesis risk

**Figure 3:**
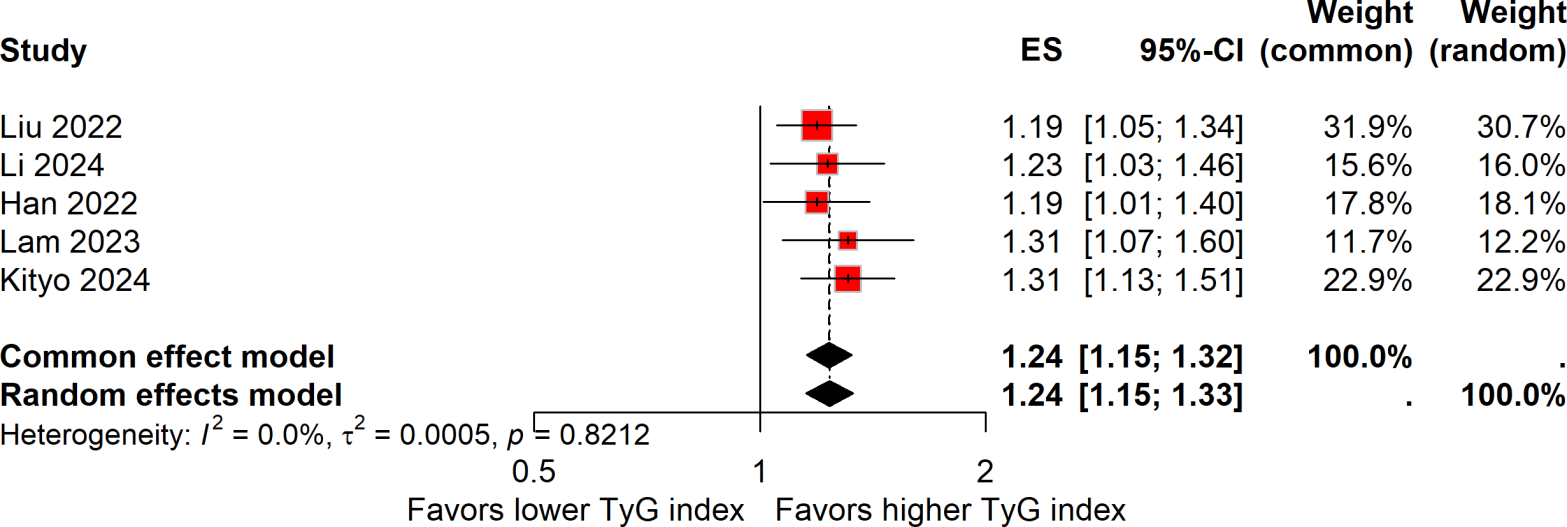
Forest plot of the association between one-unit increase in the TyG index and colorectal carcinogenesis.

**Figure 4:**
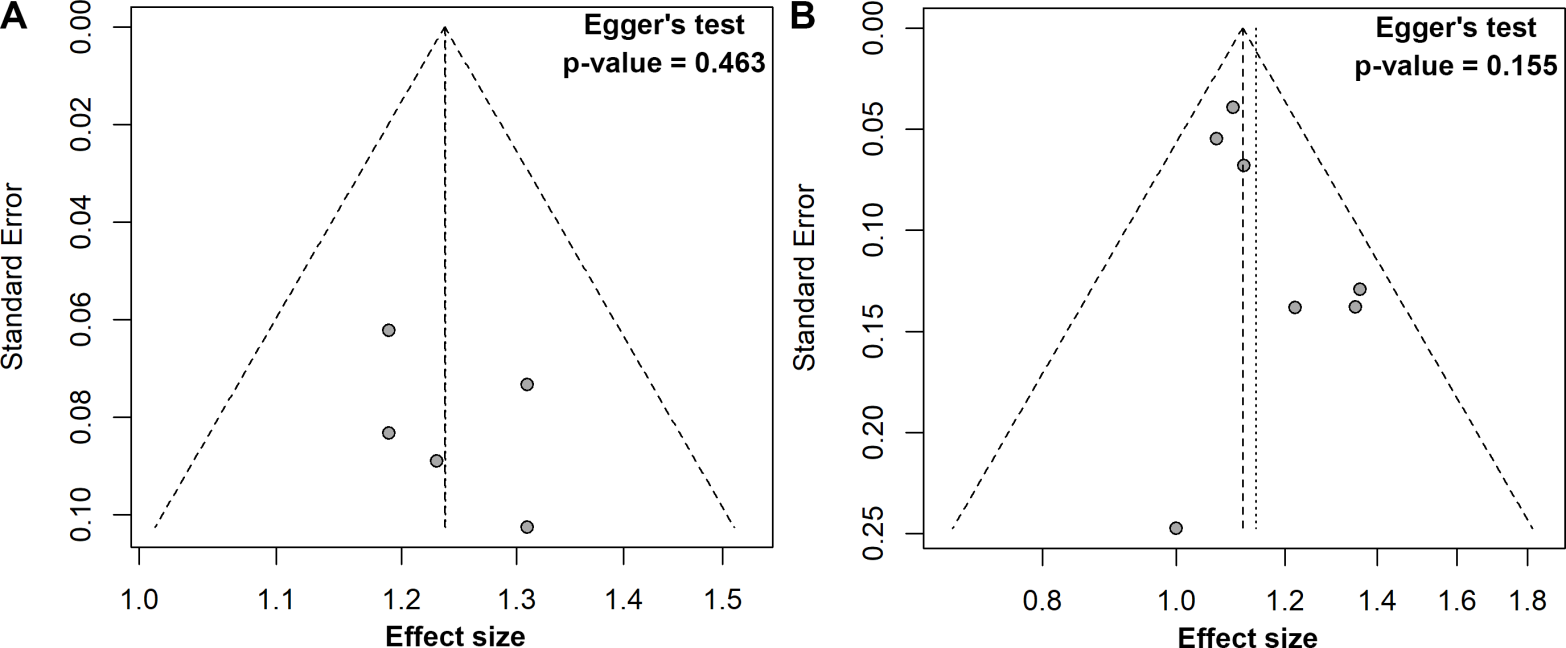
Funnel plots with Egger’s regression tests for publication bias: **(A)** one unit increase in the TyG index, **(B)** quartile 3 *vs.* quartile 1.

**Figure 5:**
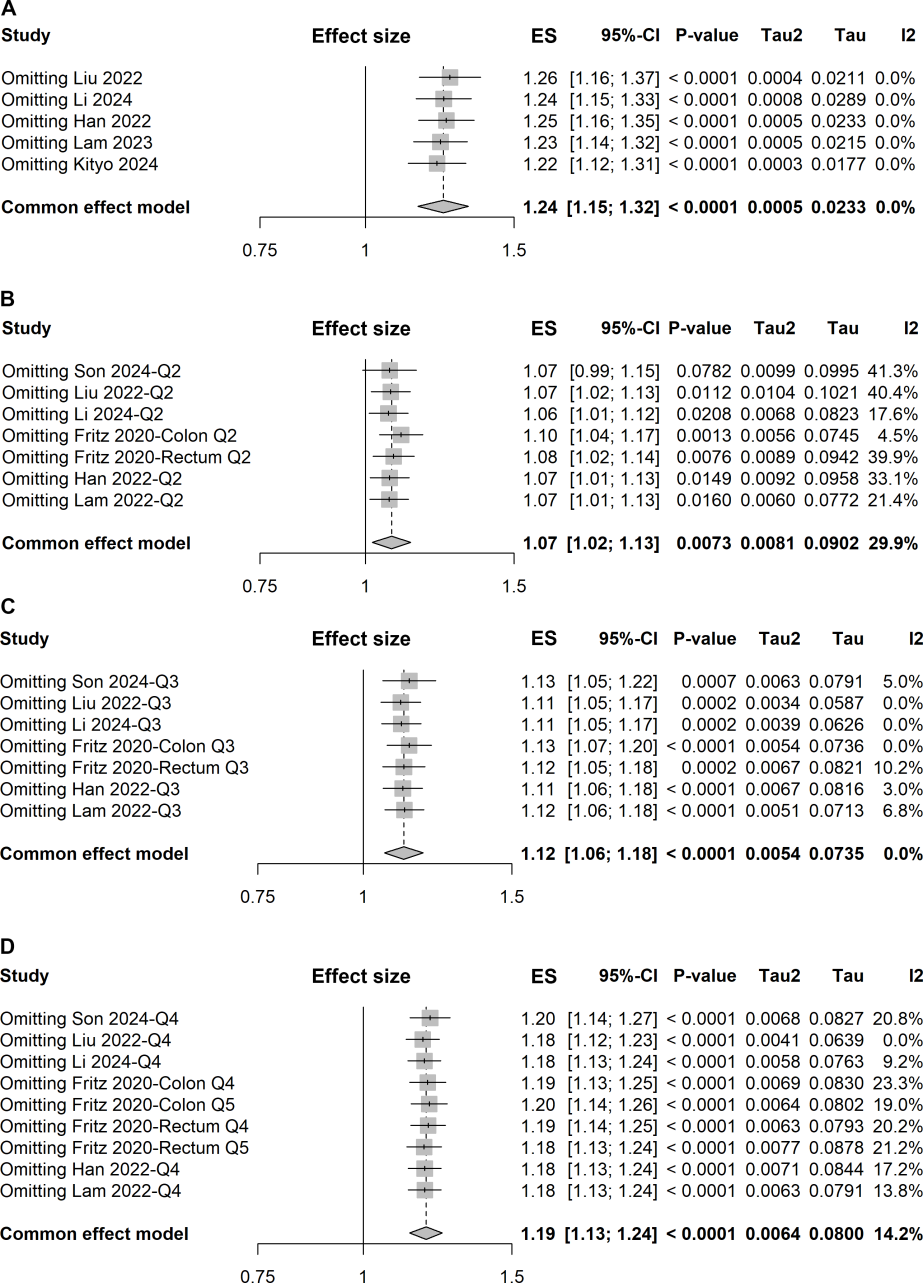
Forest plots of sensitivity analyses using the leave-one-out approach for pooled results: **(A)** one-unit increase in the TyG index, **(B)** quartile 2 *vs.* quartile 1, **(C)** quartile 3 *vs.* quartile 1, and **(D)** quartile 4 *vs.* quartile 1.

Five studies explored the association between a one-unit increment in the TyG index and the risk of colorectal carcinogenesis (12, 13, 16, 18, 19). The meta-analysis revealed that a one-unit increase in the TyG index was linked to a 24% higher risk of colorectal carcinogenesis (ES, 1.24; 95%CI, 1.15-1.32; **Figure 3**). As with the previous analysis, no heterogeneity was detected across these studies (*I²* = 0%). The funnel plot with Egger’s test suggested no significant publication bias (**Figure 4A**). The sensitivity analysis conducted via the leave-one-out method indicated that the pooled result was reliable (**Figure 5A**).

### Assessment of colorectal carcinogenesis risk across TyG index quartiles

Six studies evaluated the risk of colorectal carcinogenesis across quartiles of the TyG index (11–13, 16, 18, 19), while one study used quintiles (15). To facilitate a uniform comparison, we extracted ESs for the second quartile versus the first quartile and the third quartile versus the first quartile from all seven studies. For the comparison of the fourth quartile versus the first quartile, we included ESs from the fourth quartile of the six studies with quartile data and the fifth quartile from the study with quintile data. This method allowed us to integrate the additional data from the quintile study for the highest TyG category while maintaining a consistent framework for the other quartile comparisons.

**Figure 6:**
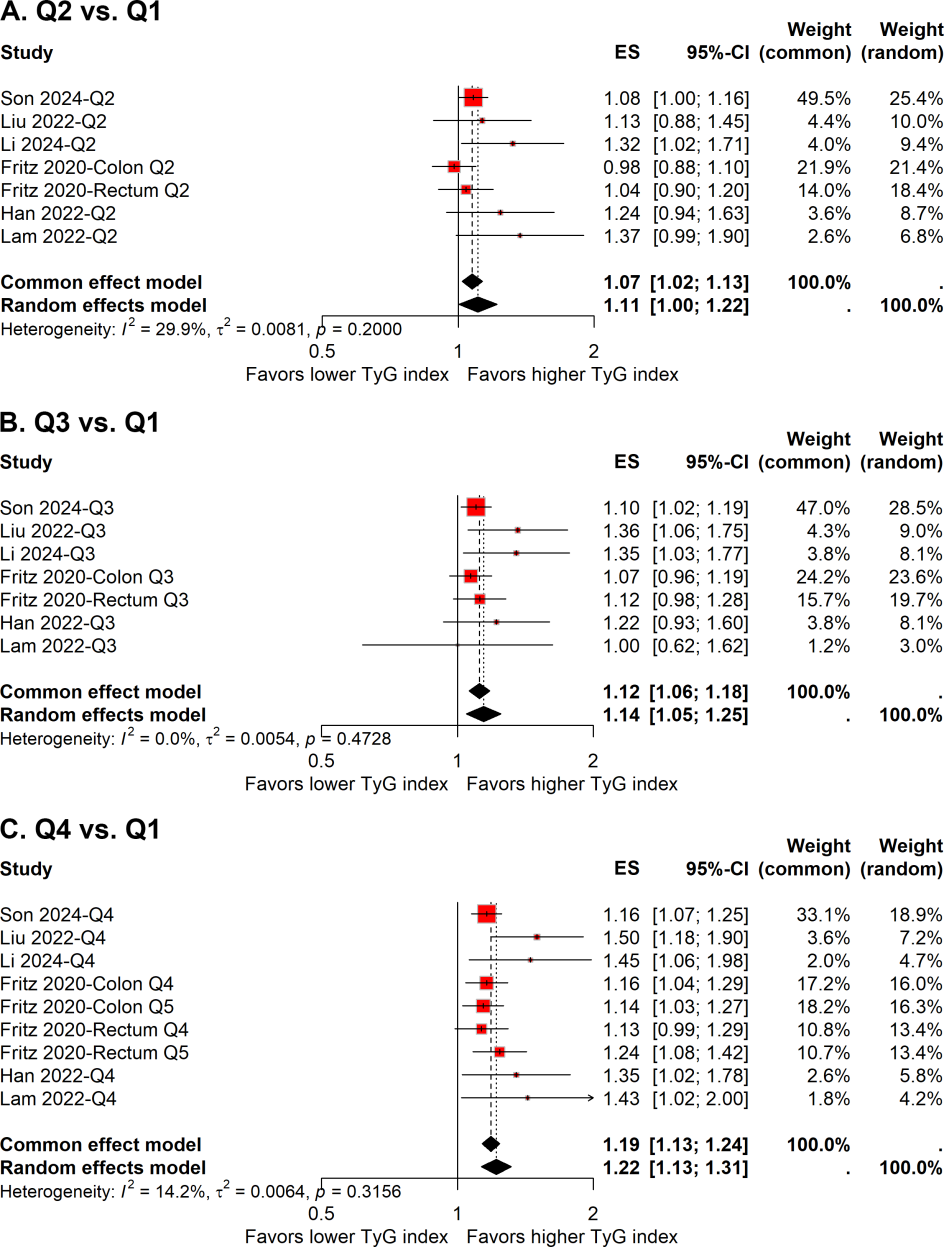
Forest plot of the association between quartile 2 vs. quartile 1 **(A)**, quartile 3 vs. quartile 1 **(B)**, and quartile 4 vs. quartile 1 **(C)** of TyG index and colorectal carcinogenesis.

Given a relatively low level of heterogeneity (*I²* = 29.9%) was observed across the studies, the pooled ES with 95% CI was determined using the fixed-effect model. The meta-analysis demonstrated that individuals in the second quartile of the TyG index had a 7% increased risk of colorectal carcinogenesis compared to the reference quartile (quartile 1) (ES, 1.07; 95% CI, 1.02-1.13; **Figure 6A**). However, the sensitivity analysis revealed that the pooled result was significantly influenced by the study conducted by Son et al. (**Figure 5B**).

In the comparison of the third quartile, no heterogeneity was present (*I²* = 0%), and the fixed-effect model was used to estimate the pooled ES. The meta-analysis suggested that individuals in the third quartile of the TyG index had a 12% higher risk of colorectal carcinogenesis than the reference quartile (ES, 1.12; 95% CI, 1.06-1.18; **Figure 6B**). The funnel plot with Egger’s test suggested no significant publication bias (**Figure 4B**), and the sensitivity analysis indicated that the pooled result was robust (**Figure 5C**).

For the fourth quartile, the meta-analysis indicated a 19% increased risk of colorectal carcinogenesis relative to the reference quartile (ES, 1.19; 95% CI, 1.13-1.24; **Figure 6C**). A low level of heterogeneity (*I²* = 14.2%) was also noted across these studies. Sensitivity analysis confirmed the robustness of the pooled result (**Figure 5D**).

## Discussion

CRC remains one of the most prevalent and deadly cancers worldwide, with an increasing burden on healthcare systems and societies (1, 2). It is well-established that lifestyle and metabolic factors play a critical role in the pathogenesis of CRC (20, 21). One of the major contributors to CRC development is IR, a condition often associated with metabolic syndrome, obesity, and diabetes (5). However, measuring IR directly is complex and costly, limiting its use in large-scale epidemiological studies. In this context, the TyG index, a surrogate marker for IR, has garnered attention due to its simplicity and ease of use. The TyG index offers several advantages, including its accessibility, simplicity, and validity. Firstly, TyG index can be easily calculated using readily available clinical data, making it a cost-effective and convenient tool for large-scale population studies and routine clinical practice. Moreover, it requires only two readily available blood tests, fasting triglycerides, and fasting glucose, avoiding the need for more complex and expensive methods like the hyperinsulinemic-euglycemic clamp technique. Additionally, it has been shown to correlate well with other established measures of IR, such as the HOMA-IR (22).

The present meta-analysis synthesizes evidence from nine observational studies involving over a million participants, revealing a significant association between the TyG index and the risk of colorectal carcinogenesis. Specifically, a continuous increase in the TyG index was associated with an elevated risk of colorectal carcinogenesis, with an ES of 1.19 (95% CI, 1.05-1.34). Moreover, a one-unit increase in the TyG index corresponded to a 24% higher risk of colorectal carcinogenesis (ES, 1.24; 95% CI, 1.15-1.32). These findings underscore the potential utility of the TyG index as a surrogate marker for predicting colorectal carcinogenesis risk. Additionally, the association between TyG index with colorectal carcinogenesis risk was also evident when comparing different quartiles of the index. Participants in the second, third, and highest quartiles exhibited significantly higher risks of colorectal carcinogenesis compared to those in the lowest quartile, with ESs of 1.07 (95% CI, 1.02-1.13), 1.12 (95% CI, 1.06-1.18), and 1.19 (95% CI, 1.13-1.24), respectively. This dose-response relationship suggests that the TyG index may serve as a valuable tool for stratifying individuals based on their risk of colorectal carcinogenesis, thereby informing personalized screening and prevention strategies.

The results of this meta-analysis align with earlier studies that have suggested a role for IR and metabolic dysfunction in colorectal carcinogenesis. This meta-analysis extends these findings by demonstrating that the TyG index, a more accessible and cost-effective alternative to HOMA-IR, can also be used to predict colorectal carcinogenesis risk. Moreover, this meta-analysis adds value to the growing body of evidence linking metabolic disorders, particularly IR, to colorectal carcinogenesis. Studies have demonstrated that insulin and glucose metabolism abnormalities contribute to CRC progression through insulin signaling pathways (23, 24). Additionally, the association between the TyG index and colorectal carcinogenesis risk observed in this meta-analysis is consistent with studies that have found a link between metabolic syndrome and increased colorectal carcinogenesis incidence (25). Studies found that individuals with metabolic syndrome had a higher risk of developing CRC (26, 27).

While our study did not directly explore the biological mechanisms underlying the observed association, several plausible pathways may explain the link between a higher TyG index and colorectal carcinogenesis. The TyG index integrates fasting triglyceride and glucose levels, thereby capturing the combined metabolic burden of hyperglycemia and dyslipidemia—two key components of insulin resistance. Elevated fasting glucose can lead to chronic hyperglycemia, which promotes oxidative stress and the formation of advanced glycation end products (AGEs). These AGEs activate inflammatory signaling cascades such as the NF-κB and STAT3 pathways, resulting in increased production of pro-inflammatory cytokines (e.g., interleukin-6 and tumor necrosis factor-alpha) that induce DNA damage, inhibit apoptosis, and promote tumor progression (28). Hyperglycemia also augments insulin and insulin-like growth factor (IGF-1) signaling, activating the PI3K/AKT/mTOR pathway, which enhances cellular proliferation, survival, and angiogenesis while suppressing antitumor immune surveillance (29, 30). In parallel, elevated fasting triglycerides reflect impaired lipid metabolism and are associated with increased circulating free fatty acids, lipotoxicity, and oxidative stress (31–33). Excess triglyceride-derived lipid intermediates can disrupt membrane lipid composition, activate the PKC and JNK pathways, and promote chronic inflammation, creating a tumor-permissive microenvironment (31–34). Dysregulated lipid and bile acid metabolism may further influence the gut microbiota, leading to dysbiosis that generates carcinogenic metabolites, disrupts epithelial barrier integrity, and enhances mucosal inflammation (35–37). Together, these component-specific processes illustrate that the TyG index is more than a surrogate marker of insulin resistance—it integrates the oncogenic consequences of both hyperglycemia and hypertriglyceridemia. This dual metabolic stress may synergistically drive epithelial proliferation, inflammation, and genomic instability, thereby contributing to colorectal tumorigenesis (4, 5).

The findings of this meta-analysis hold important implications for clinical practice and public health. The TyG index offers a simple, cost-effective, and non-invasive method for assessing IR, making it particularly suitable for large-scale epidemiological studies and routine clinical settings. Incorporating the TyG index into CRC screening protocols enables healthcare providers to identify high-risk individuals who could potentially benefit from targeted interventions, such as lifestyle modifications and pharmacological treatments to improve insulin sensitivity. Clinicians could then prioritize these individuals for more intensive screening procedures, such as colonoscopy or fecal occult blood testing, potentially leading to earlier detection of CRC and enhanced patient outcomes. Moreover, the TyG index could serve as a biomarker for monitoring the effectiveness of preventive strategies. Longitudinal studies tracking changes in the TyG index over time can help evaluate the impact of interventions on reducing CRC risk. For instance, interventions that lower the TyG index, such as weight loss programs, exercise regimens, and dietary adjustments, may be associated with decreased incidence of CRC. Additionally, public health initiatives should prioritize metabolic health education, emphasizing the TyG index as a modifiable CRC risk factor. In resource-limited settings, where advanced screening modalities are scarce, TyG-based risk algorithms could optimize resource allocation by triaging high-risk populations for priority screening.

Several limitations of this meta-analysis should be acknowledged. First, all included studies were observational in nature, precluding causal inference. Although most studies adjusted for a broad range of confounders, residual and unmeasured confounding (e.g., dietary patterns, physical activity, medication use, metabolic comorbidities, and CRC screening behaviors) may persist. Reverse causation is also plausible, as preclinical or early-stage colorectal neoplasms may influence glucose and lipid metabolism through systemic inflammation and metabolic dysregulation, thereby elevating the TyG index (38). Second, exposure assessment was heterogeneous: the TyG index was generally measured at a single baseline time point, which may not represent long-term metabolic status, and variation in fasting status, assay methodology, and study-specific cutoffs could contribute to between-study heterogeneity. Moreover, “colorectal carcinogenesis” encompassed both adenoma and carcinoma, representing different biological stages and detection probabilities; separate analyses for these entities were not feasible due to limited available data. Third, despite the lack of significant publication bias on funnel plots and Egger’s tests, small-study effects cannot be entirely excluded. Fourth, the included studies were primarily conducted in East Asian and European populations, which may limit generalizability to other ethnic groups. Fifth, the cut-off values of the TyG index varied across studies, and optimal thresholds for predicting CRC risk have not been standardized. Additionally, in our quartile-based analysis, we incorporated a study that reported data in quintiles by equating its highest quintile with the highest quartile of other studies (15). While this was a necessary and pragmatic approach to include all available data, it represents a methodological simplification that should be considered when interpreting the results. Moreover, the conclusion regarding a continuous increase in the TyG index and CRC risk is based on only two studies; while the result is statistically significant, it requires validation in a larger number of cohorts.

Future research should aim to address these methodological limitations through individual participant data meta-analyses that allow harmonized definitions of TyG exposure, consistent covariate adjustment, and evaluation of repeated TyG trajectories over time. Purpose-built prospective cohorts with standardized fasting protocols, adjudicated outcomes, and detailed colonoscopy data would further strengthen temporal inference. Dose–response analyses using restricted cubic splines could delineate whether the association is linear or threshold-dependent, while meta-regression or subgroup analyses by sex, age, BMI, diabetes status, and region may help explain residual heterogeneity. Complementary approaches such as Mendelian randomization could also provide stronger evidence for causality by assessing whether genetically predicted triglyceride–glucose profiles are associated with CRC risk independent of confounding and reverse causation. Furthermore, mechanistic and translational studies are warranted to clarify the biological underpinnings of this association. Experimental work should explore how TyG-related insulin resistance promotes colorectal tumorigenesis through insulin/IGF-1 signaling, PI3K–AKT–mTOR activation, chronic inflammation, and microbiome or bile acid alterations. Interventional studies evaluating TyG-lowering strategies—such as lifestyle modification, weight control, or pharmacologic agents including GLP-1 receptor agonists—should examine colorectal endpoints to determine whether the TyG index represents a modifiable causal determinant or a surrogate marker of metabolic risk.

## Conclusion

This meta-analysis demonstrates a significant positive association between the TyG index and the risk of colorectal carcinogenesis. The findings suggest that the TyG index could serve as a valuable surrogate marker for predicting colorectal carcinogenesis risk, offering a practical, accessible tool for identifying individuals at elevated risk. These results have important implications for CRC screening and prevention, and future research should aim to further validate the role of the TyG index in clinical practice.

## Data Availability

The original contributions presented in the study are included in the article/**Supplementary Material**. Further inquiries can be directed to the corresponding author/s.
