# Supplementary material for: Association between triglyceride-glucose index and risk of colorectal carcinogenesis: a meta-analysis of observational studies
Source: Front Oncol. 2025 Dec 17;15:1569824. doi: 10.3389/fonc.2025.1569824 (PMC12753426; doi:10.3389/fonc.2025.1569824)
Supplement: Supplementary file 1 [file DataSheet1.docx]

**Supplementary Material**

**Association between triglyceride-glucose index and risk of colorectal carcinogenesis: A meta-analysis of observational studies**

**Table S1.** Details of searching strategy.

**Table S2.** Quality assessment of included studies through the modified Newcastle-Ottawa Scale.

**Table S1.** Details of searching strategy.

| **Database** | **Search strategy** |
| --- | --- |
| ***PubMed*** | ((("Triglyceride-Glucose Index"[Title/Abstract]) OR ("Triglyceride Glucose Index"[Title/Abstract])) OR ("TyG index"[Title/Abstract])) AND ((((((("Colorectal Neoplasms"[Mesh]) OR ("Colorectal Cancer")) OR ("Colorectal Tumor")) OR ("Colorectal Carcinoma")) OR ("Colon Cancer")) OR ("Rectal Cancer")) OR ("Colorectal Adenocarcinoma")) |
| ***Scopus*** | TITLE-ABS-KEY ("Triglyceride-Glucose Index" OR "Triglyceride Glucose Index" OR "TyG index") AND TITLE-ABS-KEY ("Colorectal Neoplasms" OR "Colorectal Cancer" OR "Colorectal Tumor" OR "Colorectal Carcinoma" OR "Colon Cancer" OR "Rectal Cancer" OR "Colorectal Adenocarcinoma") |
| ***Embase*** | ('triglyceride-glucose index'/exp OR 'triglyceride glucose index' OR 'tyg index') AND ('colorectal cancer'/exp OR 'colorectal neoplasms' OR 'colorectal carcinoma' OR 'colorectal tumor' OR 'colon cancer' OR 'rectal cancer' OR 'colorectal adenocarcinoma') |
| ***Web of Science*** | (TS=(("Triglyceride-Glucose Index" OR "Triglyceride Glucose Index" OR "TyG index"))) AND (TS=("Colorectal Neoplasms" OR "Colorectal Cancer" OR "Colorectal Tumor" OR "Colorectal Carcinoma" OR "Colon Cancer" OR "Rectal Cancer" OR "Colorectal Adenocarcinoma")) |
| ***Cochrane Library*** | #1 "Triglyceride-Glucose Index" OR "Triglyceride Glucose Index" OR "TyG index" (96 items)  #2 MeSH descriptor: [Colorectal Neoplasms] explode all trees (13361 items)  #3 "Colorectal Cancer" OR "Colorectal Tumor" OR "Colorectal Carcinoma" OR "Colon Cancer" OR "Rectal Cancer" OR "Colorectal Adenocarcinoma" (25568 items)  #4 #2 OR #3 (28232 items)  #5 #1 AND #4 (2 items) |

**Table S2.** Quality assessment of included studies through the modified Newcastle-Ottawa Scale.

| **Studies** | **Selection** | | | | **Compatibility** | **Assessment** | | | **Total stars** | **Score** |
| --- | --- | --- | --- | --- | --- | --- | --- | --- | --- | --- |
|  | **Representativeness of the exposed cohort** | **Selection of the non-exposed cohort** | **Ascertainment of exposure** | **Demonstration that outcome of interest was not present at the start of study** | **Comparability of cohorts on the basis of the design or analysis*** | **Assessment of outcome** | **Was follow-up long enough for outcomes to occur** | **Adequacy of follow up of cohorts** |  |  |
| Son et al 2024 | ★ | ★ | ★ | ★ | - | ★ | ★ | ★ | 7★ | 7 |
| Liu et al 2022 | ★ | ★ | ★ | ★ | - | ★ | ★ | ★ | 7★ | 7 |
| Li et al 2024 | ★ | ★ | ★ | ★ | - (6) | ★ | ★ | ★ | 7★ | 7 |
| Choi et al 2024 | ★ | ★ | ★ | ★ | - | ★ | ★ | ★ | 7★ | 7 |
| Fritz et al 2020 | ★ | ★ | ★ | ★ | - | ★ | ★ | ★ | 7★ | 7 |
| Han et al 2022 | ★ | ★ | ★ | ★ | - (3) | ★ | ★ | ★ | 7★ | 7 |
| Okamura et al 2020 | ★ | ★ | ★ | ★ | - (3) | ★ | ★ | ★ | 7★ | 7 |
| Lam et al 2023 | ★ | ★ | ★ | ★ | - (5, 6) | ★ | ★ | ★ | 7★ | 7 |
| Kityo et al 2024 | ★ | ★ | ★ | ★ | - | ★ | ★ | ★ | 7★ | 7 |

*1. Age at diagnosis, 2. sex, 3. BMI, 4. smoking and drinking status, 5. comorbidities, and 6. family history were considered. If five to six factors of two cohorts were comparable, two stars were assigned; if three to four factors of two cohorts were comparable, one star was assigned; otherwise, no star was assigned. Studies assigned with score of seven to nine were defined as high methodological quality, while of five or six were moderate quality and of four or less were low quality. The score is equal to the total number of stars.
